# When molecular biology transforms clinical oncology: the EGFR journey in colorectal cancer

**DOI:** 10.1002/1878-0261.13754

**Published:** 2024-10-29

**Authors:** Pietro Paolo Vitiello, Nadia Saoudi González, Alberto Bardelli

**Affiliations:** ^1^ Department of Oncology University of Torino Italy; ^2^ IFOM ETS – The AIRC Institute of Molecular Oncology Milan Italy; ^3^ Vall d'Hebron Institute of Oncology (VHIO) Barcelona Spain

**Keywords:** precision medicine, colorectal cancer, EGFR, drug resistance, translational research

## Abstract

The discovery of growth factors and their involvement in cancer represents the foundation of precision oncology. The preclinical and clinical development of agents targeting epidermal growth factor receptor (EGFR) in colorectal cancer (CRC) were accompanied by big hype and hopes, though the clinical testing of such agents clashed with intrinsic and acquired resistance, greatly limiting their therapeutic value. However, a better understanding of the biology of the EGFR signaling pathway in CRC, coupled with the development of liquid biopsy methodologies to study cancer evolution in real time, fostered the clinical refinement of anti‐EGFR treatment in CRC. Such a workflow, based on the co‐evolution of biology knowledge and clinical development, allowed to couple the discovery of relevant therapy resistance mechanisms to the development of strategies to bypass this resistance. A broader application of this paradigm could prove successful and create an effective shortcut between the bench and the bedside for treatment strategies other than targeted therapy.

## Graphical abstract

The development of anti‐epidermal growth factor receptor treatment in colorectal cancer is a paradigm of how the understanding of molecular mechanisms can rapidly inform and refine the clinical use of anticancer agents. The identification of new cancer dependencies should guide the development of new treatments in parallel with the identification of the strategies to bypass acquired resistance to such treatments.

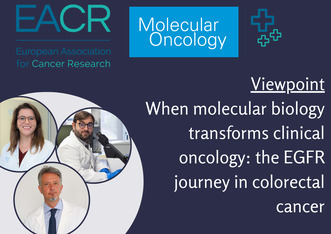

**Abbreviations**
CRC: colorectal cancer
ctDNA: circulating tumor DNA
EGF: epidermal growth factor
EGFR: epidermal growth factor receptor
NSCLC: non‐small cell lung cancer

Uncontrolled growth represents the main and most visible sign of malignancy, so much so that—unsurprisingly—*"tumor"* is the Latin word for "swelling". Yet, the biology of growth signals has become a matter of investigation only in the last six decades. We owe the discovery of growth factors as secreted molecules that directly stimulate cellular growth to Rita Levi‐Montalcini and Stanley Cohen, who were awarded the Nobel prize in Physiology and Medicine in 1986 for their discovery of the nerve growth factor (NGF) and the epidermal growth factor (EGF), respectively. In particular, the fact that an almost ubiquitously secreted factor like EGF was able to stimulate the proliferation of epithelial cells was immediately connected to a potential tumorigenic activity of this molecule and its receptor, which were shown to directly interact with the human homologs of transforming viral proteins [1]. In parallel, the extensive use of *Drosophila melanogaster* and *Caenorhabditis elegans*, two exquisitely informative model systems, allowed to better characterize the EGFR‐RAS‐MAPK signaling pathway in development and cancer growth [2]. Although it may seem obvious for modern‐day cancer scientists, the discovery that most proto‐oncogenes are involved in growth factor signaling constituted a quantum leap in advancing the understanding of tumor biology and should be regarded as a true cornerstone of molecular cancer research. As a matter of fact, the improvements of DNA cloning in the 1980s and 1990s allowed to isolate several genes related to the EGF receptor (EGFR), and to connect the dysregulation of these genes to many different cancers, including lung, breast, and gastrointestinal tract malignancies [3]. These observations made EGFR the first receptor to be proposed as a target for cancer therapy [4]. The potential mechanisms evoked by EGFR blockade ranged from the inhibition of cell cycle progression to apoptosis due to the suppression of survival signals and also included the inhibition of angiogenesis, invasion, and metastasis [4]. This plethora of anticancer activities, together with the fact that these receptors are found in many different epithelial cancers, made anti‐EGFR agents attractive candidates for the development of molecular drugs in cancer treatment [3, 4].

Colorectal cancer (CRC) constitutes a paradigm of how biological knowledge and a new generation of anticancer agents co‐evolved in the past decade. Initial clinical testing of the EGFR‐blocking antibodies cetuximab and panitumumab revealed a weak response rate of < 10% [5, 6]. Interestingly, the selection of EGFR‐overexpressing tumors was not effective in identifying colorectal cancers as susceptible of pharmacological blockade of the receptor [5]. Understanding the biology of EGFR signaling, which by then had also been resolved in mammalian cells, allowed the identification of *KRAS* activating mutations as primary responsible for intrinsic resistance to anti‐EGFR treatment in CRC [7, 8]. As outlined below in several other instances, a clinical conundrum was resolved by defining the underlying biology, and in this instance, this approach allowed to refine treatment choices by defining *KRAS* mutations as negative predictive biomarkers [7, 8].

The next clinical question to be addressed was the almost inevitable acquired resistance to anti‐EGFR. In order to unveil the biological mechanisms leading to acquired resistance, new experimental workflows were set up [9]. By crossing the sequencing data obtained from EGFR‐inhibitor‐resistant cell lines with those deriving from patients, we were able to establish that the activation of the EGFR downstream signaling pathway by *RAS* or *BRAF* mutations also constituted the main mechanism of acquired resistance to EGFR inhibitors in CRC [9, 10]. Beyond the clinical relevance, this discovery unveiled a relevant and consistent phenomenon in molecular oncology. Namely, that despite the potentially unlimited number of different evolution trajectories, cancers tend to rely on a very limited set of genetic events to reactivate crucial survival pathways and circumvent the selective pressure imposed by targeted therapy. Additionally, while the concomitant pharmacological blockade upstream and downstream of the mutated KRAS emerged as a potential strategy to achieve a complete EGFR axis inhibition, the identification of tumor‐derived mutated DNA in the bloodstream of patients allowed to monitor for the first time the genetic evolution of cancer [9, 10]. Our group became interested in the timing required by CRCs to acquire mutations in the RAS pathway under the selective pressure of anti‐EGFR agents. We discovered that mutations in the RAS pathway could be detected in circulating tumor DNA (ctDNA) up to 9 months before disease progression [9, 11]. In addition, we noticed that the levels of mutated ctDNA corresponding to resistance mutations dropped during subsequent non‐EGFR treatments which had started at the time of disease progression [11]. This notion generated a clinical window of opportunity to re‐treat patients with anti‐EGFR agents once the levels of resistance mutations had dropped in the ctDNA. This approach was successfully validated in the phase II clinical trial CHRONOS [12]. Once again, basic biology proved to be informative in the clinical management, and rechallenge treatment with anti‐EGFR agents is now considered a standard practice in the CRC continuum of care.

The pattern emerging from the first attempts to block EGFR in CRC revealed that this cancer type is addicted to EGFR‐RAS‐MAPK signaling and any perturbation to it will be likely circumvented by molecular events that are able to restore the activity of this signaling axis. Interestingly, this proved to be true not only for direct EGFR targeting but also for the blockade of activated BRAF and KRAS, whereby a combined inhibition of the deregulated oncogene and EGFR is needed to overcome primary resistance [13, 14]. Once again, how we presently use BRAF and KRAS inhibitors in the clinical setting in CRC relies on this tumors' unique biology, marking a clear difference compared to other malignancies such as melanoma or non‐small cell lung cancer [15]. Of note, the peculiar addiction of CRC to the EGFR pathway does not constitute a classical oncogene addiction, as the reliance on this pathway is not caused by an oncogenic event, but rather it reflects an intrinsic dependency embedded in the biology of the large intestine, as compared to other tumors [15].

Taking into account all of the advancements that have stemmed from the dissection of the mechanisms of anti‐EGFR activity and resistance in CRC, it is mandatory to look for applications of this research framework in the future. In other words: what have we learned from these successful investigations?

The first point regards the effective convergence of basic biology and clinical research. It was only thanks to the understanding of the unique biology of EGFR signaling that further progress in clinical targeting of this pathway was made. The integration of biological insights into therapeutic strategies represented a pivotal advancement in the fight against cancer. In this wake, new platforms are needed to facilitate the transition between preclinical research, therapeutic applications, and clinical observations to allow initiation of biology‐informed trials.

The second learning point, that is shared with most targeted therapies, is that resistance to the inhibition of oncogenic dependencies is almost inevitable and due to pathway reactivation. Interestingly, even if the EGFR‐MAPK pathway characterizes an intrinsic (lineage) oncogenic dependency rather than a classical oncogene addiction, the mechanisms of resistance to agents interfering with its dependency are redundant with the ones arising in other contexts [15]. The third point is that finding strategies to rapidly translate biological findings in the clinical setting is key to further progress. In this regard, the timely transition of biological findings into clinical advancements (and the other way around) has allowed to consolidate several key points in the treatment strategy of CRC such as: the intrinsic dependency of CRC on the EGFR axis, the role of *RAS* and *BRAF* mutations in mediating primary and acquired resistance to anti‐EGFR agents, the need for co‐inhibition of EGFR to fully suppress KRAS or BRAF oncogenic signaling, and the potential of anti‐EGFR rechallenge (Fig. 1). The development of ctDNA‐based platforms has helped to further implement this bidirectional flux of information and decreased the gap between preclinical and clinical investigations. As a matter of fact, the introduction of ctDNA‐based liquid biopsies has shortened the time required to transition from the laboratory to the patients. While it took about 45 years from the discovery of EGFR to the clinical appraisal of the first anti‐EGFR antibodies in CRC treatment, < 15 years were necessary to intercept intrinsic and acquired mechanisms of resistance, develop integrated strategies to tackle other oncogenic nodes along the pathway, and tailor rechallenge strategies based on the circulating levels of mutations responsible for resistance.

**Fig. 1:**
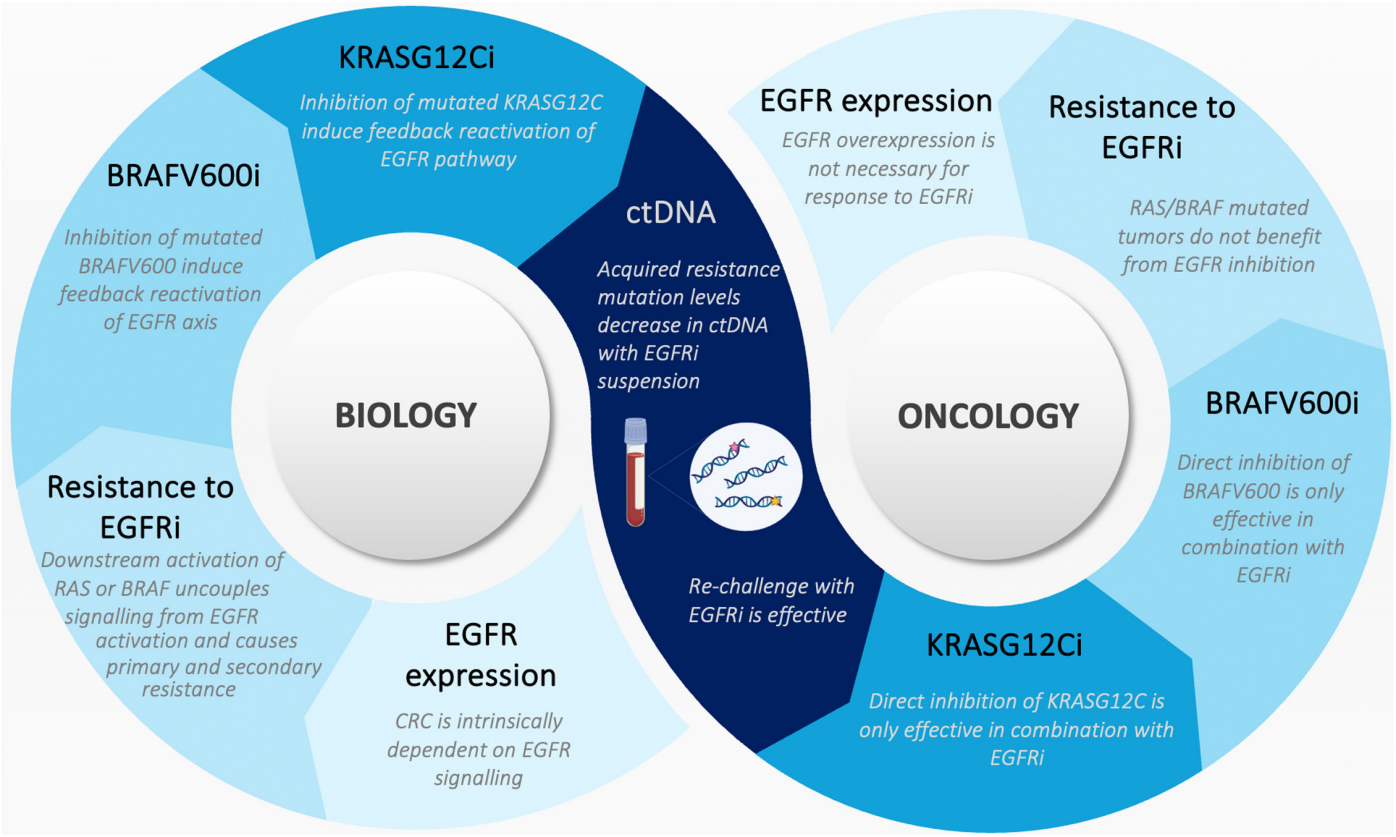
Crosstalk between biological findings and clinical achievements in targeting the epidermal growth factor receptor (EGFR) axis in colorectal cancer treatment. BRAFV600i, BRAFV600 inhibitor; ctDNA, circulating tumor DNA; EGFRi, epidermal growth factor receptor inhibitor; KRASG12Ci, KRASG12C inhibitor.

Indeed, the integration of biological knowledge to the design of observational and investigational clinical trials is expected to not only expedite the clinical development but also to direct resources toward treatment strategies that are more likely to be clinically impactful. In conclusion, since Stanley Cohen firstly came across EGF while investigating biological extracts able to induce an early eye opening in mice pups [16], we can conclude that understanding EGFR biology has been an eye‐opening endeavor for more than 60 years, and probably for the next to come.

## Conflict of interest

PPV served in a consulting role for Biocartis and has received speaking fees from Biocartis and Merck outside of the current manuscript. NSG has received speaking fees from Amgen outside of the current manuscript. AB served in a consulting/advisory role for Guardant Health and Inivata. AB received research support by Neophore, AstraZeneca and Boehringer outside of the current manuscript. AB is cofounder and shareholder of NeoPhore limited. AB is a shareholder of Kither. AB is a member of the scientific advisory board of NeoPhore, Inivata, and Roche/Genentech.

## Author contributions

PPV and AB conceived the manuscript. PPV and NSG wrote the manuscript. PPV, NSG and AB revised the manuscript. All the authors read and approved the final manuscript.
